# Hypoxia-Associated Changes in Striatal Tonic Dopamine Release: Real-Time *in vivo* Measurements With a Novel Voltammetry Technique

**DOI:** 10.3389/fnins.2020.00869

**Published:** 2020-08-18

**Authors:** Abhijeet S. Barath, Aaron E. Rusheen, Juan M. Rojas Cabrera, J. Blair Price, Robert L. Owen, Hojin Shin, Dong Pyo Jang, Charles D. Blaha, Kendall H. Lee, Yoonbae Oh

**Affiliations:** ^1^Department of Neurologic Surgery, Mayo Clinic, Rochester, MN, United States; ^2^Medical Scientist Training Program, Mayo Clinic, Rochester, MN, United States; ^3^Mayo Clinic Alix School of Medicine, Mayo Clinic, Rochester, MN, United States; ^4^Department of Biomedical Engineering, Hanyang University, Seoul, South Korea; ^5^Department of Biomedical Engineering, Mayo Clinic, Rochester, MN, United States

**Keywords:** hypoxia, striatum, rodent, tonic dopamine, voltammetry

## Abstract

**Introduction:**

Striatal tonic dopamine increases rapidly during global cerebral hypoxia. This phenomenon has previously been studied using microdialysis techniques which have relatively poor spatio-temporal resolution. In this study, we measured changes in tonic dopamine during hypoxia (death) in real time with high spatio-temporal resolution using novel multiple cyclic square wave voltammetry (MCSWV) and conventional fast scan cyclic voltammetry (FSCV) techniques.

**Methods:**

MCSWV and FSCV were used to measure dopamine release at baseline and during hypoxia induced by euthanasia, with and without prior alpha-methyl-p-tyrosine (AMPT) treatment, in urethane anesthetized male Sprague-Dawley rats.

**Results:**

Baseline tonic dopamine levels were found to be 274.1 ± 49.4 nM (*n* = 5; mean ± SEM). Following intracardiac urethane injection, the tonic levels increased to a peak concentration of 1753.8 ± 95.7 nM within 3.6 ± 0.6 min (*n* = 5), followed by a decline to 50.7 ± 21.5 nM (*n* = 4) at 20 min. AMPT pre-treatment significantly reduced this dopamine peak to 677.9 ± 185.7 nM (*n* = 3). FSCV showed a significantly higher (*p* = 0.0079) peak dopamine release of 6430.4 ± 1805.7 nM (*n* = 5) during euthanasia-induced cerebral hypoxia.

**Conclusion:**

MCSWV is a novel tool to study rapid changes in tonic dopamine release *in vivo* during hypoxia. We found a 6-fold increase in peak dopamine levels during hypoxia which was attenuated with AMPT pre-treatment. These changes are much lower compared to those found with microdialysis. This could be due to improved estimation of baseline tonic dopamine with MCSWV. Higher dopamine response measured with FSCV could be due to an increased oxidation current from electroactive interferents.

## Introduction

Transient hypoxic ischemia or hypoxic ischemia following death results in massive striatal dopamine release within minutes (Phebus et al., 1986; Ahn et al., 1991). Hypoxic ischemia has been shown to cause loss of vulnerable dopaminergic neuronal cells in the substantia nigra and ventral tegmental area of human neonates and rat pups (Burke et al., 1992; Pagida et al., 2013). Excess dopamine released in response to hypoxia exerts neurotoxic effects (Murphy et al., 1996; Zhang et al., 1998). This is preventable to some degree by dopamine depletion and drugs which exert neuroprotective effects on dopaminergic neurons (Clemens and Phebus, 1988; McPherson and Juul, 2008). In addition, the hypoxia sensory neurons, glomus type I cells of the carotid body, release dopamine and other neurotransmitters in response to hypoxia (Toledo-Aral et al., 2002; Carroll and Kim, 2005). Another interesting observation is a blunted response to hypoxia and impaired perception of dyspnea in patients with Parkinson’s disease and individuals who have had bilateral carotid body removal (Lugliani et al., 1971; Onodera et al., 2000). These observations suggest an intimate relationship between hypoxia and dopaminergic neurons.

Massive striatal dopamine release associated with ischemia and death has previously been shown using *in vivo* microdialysis and voltammetry (Phebus et al., 1986; Gonzalez-Mora et al., 1989). Microdialysis has the capability of analyzing tonic levels of several analytes simultaneously. However, measurements can typically be performed only once every 10 min and require offline analyte separation and analysis techniques (Chefer et al., 2009). Voltammetry on the other hand is capable of providing sensitive, on-line real-time measurement of oxidizable analytes such as dopamine, serotonin, and ascorbate. However, most voltammetry techniques are limited by poor selectivity and cannot measure tonic (basal) levels of these analytes (Oh et al., 2016).

Recently, we have described a novel voltammetry technique called multiple-cyclic square wave voltammetry (MCSWV) (Oh et al., 2018) to examine tonic dopamine release *in vivo*. MCSWV allows sensitive, real-time measurements of tonic (basal) dopamine levels with a high spatio-temporal resolution and displays improved selectivity over conventional background subtraction voltammetry techniques, such as fast scan cyclic voltammetry (FSCV). In this study we used MCSWV to measure the rapid changes in tonic dopamine levels following cardio-pulmonary arrest with intracardiac urethane injection. We also present a case of dopaminergic changes during spontaneous intraoperative hypoxia and subsequent resuscitation in an anesthetized animal.

## Materials and Methods

### Experimental Animals

Male Sprague Dawley rats (Envigo, United States) weighing between 250 and 360 grams (age 8–13 weeks) were used for this study. They were housed in pairs, and maintained in standard size cages with a 12-h light-dark cycle (lights on at 0600 h) in an AAALAC accredited vivarium (21°C, 45% humidity). Food and water were available *ad libitum*. Animals from two studies, primarily designated to investigate the mechanism of deep brain stimulation are included in this report. These studies were approved by the Institutional Animal Care and Use Committee (IACUC), Mayo Clinic, Rochester. The NIH Guide for the Care and Use of Laboratory Animals guidelines (Department of Health and Human Services, NIH publication No. 86-23, revised 1985) were followed for all aspects of animal care. Here, we focus on the observations made upon euthanizing the rodents at the end of these studies.

### Electrodes

Carbon fiber microelectrodes (CFMs) were prepared using standard methods described previously (Clark et al., 2010; Oh et al., 2016). T300 carbon fibers (Cytec carbon Fibers LLC, Greenville, SC, United States) were used in the manufacturing of CFMs for FSCV studies. AS4 carbon fibers (Hexel, Stamford, CT, United States) were used in the manufacturing of CFMs for MCSWV studies. The AS4 carbon fibers were electrically deposited with a mixture of poly(3,4-ethylenedioxythiophene) and Nafion to increase their sensitivity and selectivity to dopamine and reduce *in vivo* biofouling (Vreeland et al., 2015).

### Surgery

The animals were anesthetized with urethane (1.5 g/kg i.p.; Sigma-Aldrich, St Louis, MO, United States) and administered buprenorphine (0.05–0.1 mg/kg s.c., Par Pharmaceutical, Chestnut Ridge, NY, United States) for analgesia. Following anesthesia, they were immobilized on a stereotactic frame (David Kopf Instruments, Tujunga, CA, United States) with a rat incisor bar set at −3.3 mm DV. The skin overlying the animal’s head was shaved and an incision was made to expose the skull. A trephine bit was used to drill holes in the skull for placement of the CFM and stimulating electrodes. The CFM was placed in the dorsal striatum (AP: + 1.2 mm, ML + 2 or + 3 mm, DV between 4 and 5 mm below skull surface) (Paxinos and Watson, 2007). Concentric bipolar stimulation electrodes were placed in deep brain nuclei being studied. An Ag/AgCl reference electrode was placed in the cortex of the contralateral hemisphere. All stereotactic coordinates were defined with respect to bregma. The breath rate of animals was monitored continuously with a RespiRat device (Intuitive Measurement Systems, AZ, United States).

### Study Groups and Voltammetry Recordings

The animals were divided into two groups as described below. This report discusses the changes in dopamine levels observed in the immediate period after euthanizing the animals with intracardiac (IC) injection of 1 mL urethane (280 mg/mL). The depth of anesthesia was assessed using the toe pinch maneuver to elicit reflex withdrawal of the limb to pain before administering euthanasia. Absence of the withdrawal reflex indicated deep anesthesia. This helped minimize distress to the animal from IC injection.

Group 1: CFMs were implanted into the dorsal striatum and FSCV was performed to measure phasic dopamine release in response to electrical stimulation. Dopamine release during global cerebral hypoxia was recorded for several minutes after euthanasia. A triangular waveform with a holding potential of -0.4 V, peak potential of 1.3 V, scan rate of 400 V/s and a scanning frequency of 10 Hz was used for FSCV. The recordings were performed with WINCS Harmoni device and controlled using WincsWare in Harmoni software (Mayo Clinic, Rochester, MN, United States) (Lee et al., 2017). After experimentation, dopamine levels were determined by calibration of CFMs with 1 μM dopamine solution using a flow cell injection apparatus.

Group 2: CFMs were implanted in the dorsal striatum with respect to bregma: AP + 1.2, ML + 3, and DV −4 to −5 mm and tonic dopamine levels were then recorded with MCSWV (Oh et al., 2018). After a baseline measurement for 60–90 min the animals were euthanized and MCSWV recordings were continued for 15–25 min post-euthanasia. We used MCSWV parameters as previously described (Oh et al., 2018). Briefly, five cyclic square waveforms, each consisting of square wave oscillations superimposed on a symmetric staircase waveform were applied in tandem at a frequency of 0.1 Hz. We fixed the waveform parameters, E_Staircase_, E_Initial_ (= E_End_) and τ at 25 mV, −200 mV, and 1.0 ms, respectively. The technique was implemented using a commercial electronic interface (NI USB-6363, National Instruments) with a base-station PC and in-house software written in LabVIEW 2016 (National Instruments, Austin, TX). Data was processed using MATLAB (MathWork Inc., Natick, MA, United States). Dopamine levels were determined by calibration of CFMs with 1 μM dopamine solution after the experiment. Quadratic regression described previously was used to determine the tonic dopamine concentration (Oh et al., 2018). All results are expressed as mean ± SEM.

Group 3: These animals were treated similar to group 2, except that pharmacological manipulation was performed to assess the contribution of newly synthesized dopamine pool to dopamine release post-euthanasia. Alpha-methyl-p-tyrosine (AMPT), a catecholamine synthesis inhibitor (which acts via competitive antagonism of the enzyme tyrosine hydroxylase), was administered at a dose of 250 mg/kg i.p. at 90–120 min before IC urethane injection. For clarity, group 3 will be referred to as AMPT group and group 2 as non-AMPT group further in the manuscript.

### Statistics

Non-parametric Mann-Whitney test was used to compare the means between following data sets – (a) peak dopamine release following euthanasia-induced cerebral hypoxia measured with FSCV vs. MCSWV (group 1 vs. group 2), and (b) peak dopamine release between animals treated vs. those not treated with AMPT (group 2 vs. group 3).

## Results

### Phasic Dopaminergic Changes With Hypoxia

**FIGURE 1:**
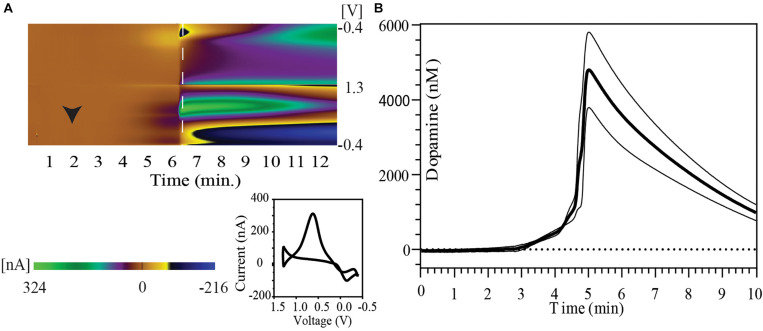
Striatal dopamine release in response to euthanasia-induced hypoxia measured with FSCV. **(A)** Pseudo-color plot shows time trend of change in oxidation and reduction current after intracardiac urethane injection (black arrowhead) in a representative animal. A shift in background current can be seen in later part of pseudo-color plot. The 2-D voltammogram inset shows dopamine oxidation and reduction at the time point indicated by dashed white line on the pseudo-color plot. **(B)** Concentration vs. time plot show phasic dopamine changes after intracardiac urethane injection (bold line represents mean concentration of dopamine over time; thin black lines represent SEM). The point of highest observed dopamine peak response was used to align the time trends.

Immediately following IC urethane injection there was rapid cessation in respiration and cardiac activity. Figure 1A shows a FSCV pseudo-color plot showing the time course of phasic dopaminergic changes following urethane injection in a representative animal. Immediately after the injection, a gradual increase in the oxidation signal around + 0.6 V was observed followed by a rapid spike within 6 min of euthanasia. The dopamine oxidation voltage shifted to more positive values, while the dopamine reduction voltage shifted to more negative values during the time course of this event. Other changes were also observed in background currents during further course of time correlating to a drift in dopamine redox potentials. The inset image shows a 2-D voltammogram for dopamine oxidation and reduction at the peak of the dopamine response.

A peak dopamine release of 6430.4 ± 1805.7 nM dopamine with a range of 2523.1–12942.5 nM (*n* = 5 rats) was observed in this series. Five animals were used to determine the peak dopamine response with death. However, the recording was discontinued shortly after the dopamine peak in one of the animals. Therefore, data from only four animals with at least 5 min of recording post-dopamine peak was used to construct the figure. Figure 1B shows the temporal pattern of change in extracellular dopamine for these four animals. Their time course has been aligned by the peak of their dopamine response.

### Tonic Dopaminergic Changes With Hypoxia

**FIGURE 2:**
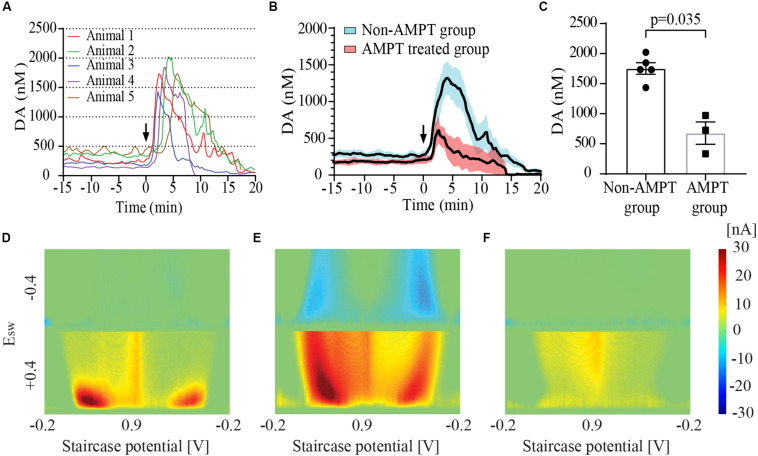
Striatal dopamine (DA) release in response to euthanasia-induced hypoxia measured using multiple cyclic square wave voltammetry (MCSWV). **(A)** Tonic dopamine levels rise rapidly following intracardiac (IC) urethane injection [black arrows in **(A)** and **(B)**] reaching a peak concentration of about 6 times the basal values within 3–4 min, followed by a decline to less than 20% of the baseline concentration by 20 min (*n* = 5 rats for time point 0–17 min, *n* = 4 rats from 17–20 min). In most animals, levels gradually declined over a 20 min period following the peak. However, in animal 4, the levels became undetectable at around 8 min following IC injection. **(B)** Changes in tonic dopamine levels observed in animal groups with and without alpha-methyl-p-tyrosine (AMPT) pretreatment. Bold line represents mean concentration of dopamine over time; red and blue shaded areas represent the SEM for the respective groups. **(C)** Pretreatment of animals with AMPT lead to a significant decrease in dopamine release (*p* = 0.035) observed post-euthanasia. Snapshots of pseudo-color plots during **(D)** baseline tonic recording, **(E)** peak oxidation signal response, and **(F)** 20 min after urethane injection in a representative animal from **(A)**.

Figure 2A show the time course of dopamine concentration at baseline and following IC injection in five animals (Group 2). These baseline tonic concentrations of dopamine yielded a mean value of 274.1 ± 49.4 nM (*n* = 5 rats). Figure 2D shows the MCSWV pseudo-color plot recorded at baseline in a representative animal. The Y axis of the plot represents the square wave potential with the lower half representing positive potential and the upper half representing negative potential. The X axis represents the staircase potential which increases from –200 mV (E_Initial_) to + 900 mV (E_peak_) and back to –200 mV (E_End_) during a cycle lasting 90 ms. Five such cycles occur consecutively and the final voltammogram is obtained by subtracting the results of the 2nd cycle from 5th cycle. The third dimension of the pseudo-color plot is the oxidation current, visualized with a color-scale gradient. The fourth dimension of the pseudo-color plot is time which is captured in a video film. The oxidation peaks are seen in red while the reduction peaks are visualized in blue. Two peaks each for oxidation and reduction are seen with MCSWV. The oxidation and reduction peak on the left occur during ramp up of the staircase potential. The peaks on the right occur during ramp down of staircase potential. The oxidation peak on the left was found to be most sensitive for detecting the dopamine signal as it had the highest current value. This peak was used for determining dopamine concentrations.

Following IC urethane injection, the tonic concentration of dopamine rapidly increased to a mean peak value of 1753.8 ± 95.7 nM (*n* = 5 rats). The post-mortem dopamine release measured with MCSWV was significantly lower than that measured with FSCV (*p* = 0.0079). The pseudo-color plot in Figure 2E shows the peak dopamine oxidation and reduction signal seen during euthanasia-induced global cerebral hypoxia in one representative animal. A shift in the oxidation potential for dopamine was observed similar to that found with FSCV. The reduction peaks are seen more prominently, especially during ramp down of the applied staircase potential due to the relatively large increase in dopamine release. The time to peak dopamine oxidation after injection was found to be 3.6 ± 0.6 min (*n* = 5 rats). At 15 min post-injection, dopamine levels were found to be 204.2 ± 72.2 nM (*n* = 4 rats), dropping to 50.7 ± 21.5 nM (*n* = 4) at 20 min. Figure 2F shows the low dopamine oxidation signal seen 20 min after the IC urethane injection in a representative animal. Most recordings were discontinued between 15 and 25 min following the injection. AMPT pre-treatment lead to a significantly lower post-mortem peak dopamine release of 677.9 ± 185.7 nM (*n* = 3), compared to animals who were not treated (*p* = 0.035) as seen in Figures 2B,C).

### Spontaneous Intra-Operative Hypoxia and Air-Way Clearance

**FIGURE 3:**
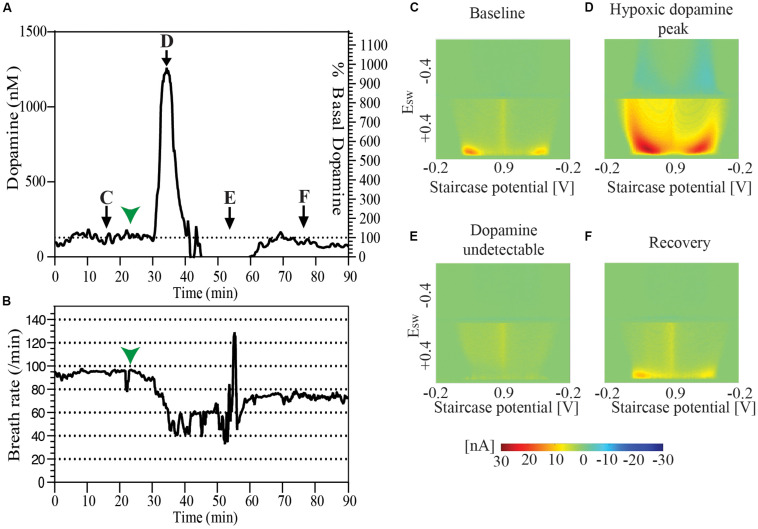
**(A)** Changes in tonic dopamine release accompanying spontaneous drop of breath rate and labored respiration followed by recovery in a single animal. **(B)** Record of breath rate (running averaged over 30 s) during the same period as indicated in **(A)** shows a decrease in breath rate starting 7–8 min before the observed increase in the dopamine oxidation signal signal marked by green arrowheads in both **(A)** and **(B)**. Snapshots of pseudo-color plots from different time points indicated in **(A)**. **(C)** Baseline tonic recording, **(D)** peak of dopamine oxidation signal, **(E)** post-episode undetectable dopamine, and **(F)** recovery of dopamine response.

During the course of one of our experiments an animal spontaneously developed labored breathing with decreased breath rate. Oral and respiratory suctioning was performed with a thin flexible polyethylene tube attached to a syringe with the events noted in the experiment log. Analysis of data revealed a baseline tonic dopamine level of 128.4 nM which spiked to 1261.1 nM during the course of the presumed spontaneous hypoxic episode. Dopamine was undetectable at 10 min after the peak, and could be detected again at 20 min. Tonic dopamine levels were re-established at 94.8 nM, 30 min after the episode. During this period average respirations per minute fell from baseline of ∼90 respirations per minute to ∼50 for a period of 20–25 min, beginning about 7–8 min before the peak of the presumed hypoxic-induced dopamine response. It returned to the mid-70 respirations per minute after successful airway clearance (Figure 3).

## Discussion

### Tonic Dopamine Increase in Global Cerebral Hypoxia Encountered During Death

We have shown that the massive dopamine release occurring with euthanasia-induced global cerebral hypoxia can be quantified in real time with high spatial (≈50 μm) and temporal resolution (≈10 s) using MCSWV. While this phenomenon has been extensively reported in the literature, to the best of our knowledge this is the first report describing real-time *in vivo* measurement of changes in tonic levels of extracellular dopamine due to hypoxia. In our study, the baseline tonic dopamine concentration in the dorsal striatum of urethane anesthetized male rats was found to be 274.1 ± 49.4 (*n* = 5 rats). Following cardiac injection of urethane, tonic levels of dopamine peaked at 1753.8 ± 95.7 nM (*n* = 5 rats), showing an average 6-fold increase over baseline (Figures 2A,B). Previous studies using microdialysis have found a 200–400 fold increase in striatal dopamine at death (Vulto et al., 1988; Gonzalez-Mora et al., 1989). Microdialysis probes inherently cause damage to dopamine terminal release sites due to their relatively large size (Chefer et al., 2009). It has been proposed that a trauma layer surrounding the microdialysis probe suppresses dopamine release and that re-uptake removes much of the dopamine diffusing toward the probe leading to an underestimation of tonic extracellular concentrations (Chefer et al., 2009). Upon death, the reuptake mechanism of dopamine is disrupted and consequently, the flux of dopamine into the dialysate increases disproportionately compared to normal physiological conditions. Thus, the ratio of post-mortem to physiological tonic concentration is several folds higher. In part, this may account for the discrepancy seen in the percent change in tonic dopamine concentration with our study compared to microdialysis. Also, we observed a discrepancy between peak dopamine levels estimated with FSCV and MCSWV. There are several possible reasons for this. These include potentially poor selectivity of FSCV with mixed release of electroactive interferents during death and background drift.

Following euthanasia, the rise in tonic dopamine levels is very rapid taking on average 3.6 min to reach a peak concentration from baseline. This is in line with two previous studies which have reported a 5–6 min period for dopamine levels to reach a peak following euthanasia (Phebus et al., 1986; Vulto et al., 1988). Ahn et al. (1991) have reported that critical events related to the dopamine surge occur between 2 and 10 min after ischemia in gerbils with unilateral carotid ligation (Ahn et al., 1991). These release dynamics cannot be effectively studied using microdialysis given its relatively limited spatial (1–2 mm by 0.2 mm probe dimensions) and temporal resolution (≥ 1 min, more often around 10 min) (Chefer et al., 2009). As demonstrated in this work, voltammetry techniques are better suited to study the rate of increase in dopamine oxidation signal as they permit high spatio-temporal resolution.

Dopamine within dopaminergic neurons exists in readily releasable and reserve storage pools. These pools are differently sensitive to various pharmacological manipulations. We used AMPT to deplete dopamine in the newly synthesized, readily releasable pool (Thomas et al., 2008). Following this we observed a significant reduction in post-mortem dopamine release, although it was not completely abolished. This is likely due to the fact that AMPT has little effect on the reserve pool of dopamine which also partakes in dopamine release post-mortem.

### Mechanisms of Dopaminergic Response in Hypoxia

Given the well-established evidence for the sensitivity of dopaminergic neurons to hypoxia and previous studies demonstrating this phenomenon in both a hypoxia model, as well as with death, strongly suggest hypoxia as the likely explanation for massive dopamine release observed in our study (Phebus et al., 1986; Gonzalez-Mora et al., 1989; Ahn et al., 1991). Three primary mechanisms are thought to increase dopamine extracellular concentrations during hypoxia: (1) increased release, (2) impaired reuptake, and (3) extracellular volume contraction. Following hypoxia, there is rapid increase in levels of extracellular potassium, reaching up to 60 mM within 3–4 min (Hansen, 1985). Increased extracellular levels of potassium have been shown to increase dopamine release (Imperato and Di Chiara, 1984). In addition, dopamine reuptake is an ATP dependent process (Cooper et al., 2003) and ischemia leads to depletion of ATP within 5 min of onset resulting in impaired dopamine reuptake (Munekata and Hossmann, 1987). Furthermore, anoxic depolarization of neurons and glia by ischemia (Jarvis, 2001) leads to a ∼50% reduction in extracellular volume within minutes (Rice and Nicholson, 1987). This phenomenon, in turn, may contribute to the rise in dopamine concentration seen after onset of ischemia.

### Dopamine Increases Reversibly During a Brief Episode of Hypoxia

We observed a sharp spontaneous rise in dopamine in one of our animals coinciding with an episode of a decrease in respiration rate and labored breathing (Figure 3). After this episode, dopamine was undetectable for a period of time, followed by reappearance of tonic dopamine albeit at lower levels then before the episode. It has been shown previously that a brief period of hypoxia leads to a sharp rise in striatal dopamine concentration which declines back to baseline following resumption of circulation (Ahn et al., 1991; Akiyama et al., 1991). Thus, it is likely that the observed decrease in respiration and consequent hypoxia results in this sharp rise in dopamine. The lower levels in tonic dopamine following this episode may reflect a depletion of dopamine stores or injury to a subset of dopaminergic neurons with a resultant loss of function. A previous microdialysis study has shown that following hypoxia, dopamine levels return to pre-hypoxia baseline values (Akiyama et al., 1991). However, it is difficult to draw direct comparison between that study and our observation due to differences in the origin and timing of hypoxia, sample size, and the uncontrolled nature of the hypoxic episode in our study.

The phenomenon of massive dopamine release in hypoxia followed by resumption of tonic levels drew our attention to a little discussed clinical and forensic problem, namely auto-erotic asphyxiation (AEA) behaviors (Quinn and Twomey, 1998; Bunzel et al., 2019). Increase in striatal and nucleus accumbens dopamine transmission has been shown during sexual activity (Pfaus et al., 1990, 1995). Drawing parallels, this indicates that the mechanism of AEA derived gratification may involve a sharp dopamine spike, akin to sexual activity and addiction (Baik, 2013). Unfortunately, a number of AEA cases come to light only following accidental deaths during this behavior (Pfaus et al., 1990, 1995). Studying the role of dopamine and possibly drugs targeting dopamine release in hypoxia may have therapeutic value for these patients. Another clinical situation where transient hypoxia is encountered is syncope and cardiac arrests. Ten percent of patients with cardiac arrest (Van Lommel et al., 2001) and 60% patients experiencing syncope (Lempert et al., 1994) report visual and auditory hallucinations which are similar to those described in near-death experiences (Nelson, 2015). Healthy volunteers exposed to transient cerebral hypoxia by induction of syncope commonly reported a feeling of weightlessness, detachment and peace which some compared to previous drug or meditation experience (Lempert et al., 1994). These experiences may be mediated, in part, by dopamine release seen with transient hypoxia. However, it is likely hypoxia-induced release of other neurotransmitters, such as serotonin, also play a role in these phenomena (Wutzler et al., 2011).

### Neurotoxicity of Dopamine

Dopamine release in hypoxia is known to have neurotoxic effects on dopaminergic, as well as other striatal neurons (Murphy et al., 1996; Zhang et al., 1998). Animal studies have shown that depletion of dopamine has a neuroprotective effect on striatal neurons exposed to hypoxia (Clemens and Phebus, 1988; Buisson et al., 1992). AMPT, a reversible tyrosine hydroxylase inhibitor, has also been shown to reduce methamphetamine associated dopaminergic neurotoxicity (Thomas et al., 2008). This raises the possibility of a neuroprotective strategy with reversible dopamine depletion in cardiac arrest. Such an approach, if successful in animal models, may find future clinical applications. MCSWV could serve as a reliable tool to study dopamine release dynamics in such studies.

## Conclusion

In conclusion, we have demonstrated MCSWV as a novel tool to study real-time rapid changes in tonic dopamine dynamics during hypoxia. This conclusion is supported by the observations we have made during euthanasia-induced hypoxia, which are in line with previous studies utilizing microdialysis and FSCV. We found that following euthanasia with IC urethane injection, there is a 6-fold rise in tonic dopamine concentrations which declines to less than 20% of the baseline concentration at 20 min. This post-mortem dopamine release is reduced by depletion of newly synthesized, readily releasable dopamine pool using AMPT. The high spatial and temporal resolution of this technique makes it an ideal tool to investigate dopaminergic release dynamics involved in the mechanism of deep brain stimulation, drugs of abuse, motivated behaviors, and pharmacological agents targeting dopaminergic systems.

## Data Availability Statement

All datasets generated for this study are included in the article/supplementary material.

## Ethics Statement

The animal study was reviewed and approved by the Institutional Animal Care and Use Committee (IACUC), Mayo Clinic, Rochester.

## Author Contributions

KL, YO, CB, and DJ conceptualized the study. AB, AR, JR, and JP conducted the experiments and collected the data. AB, YO, DJ, and HS designed the analyses. AB conducted the analyses. AB, AR, KL, CB, YO, and HS wrote the manuscript. KL and YO supervised all aspects of this work. AB and RO drafted the figures. All authors commented on and accepted the final version of the manuscript.

## Conflict of Interest

The authors declare that the research was conducted in the absence of any commercial or financial relationships that could be construed as a potential conflict of interest.
